# Treatment of Rectal Fistula

**Published:** 1918-10

**Authors:** Charles J. Drueck

**Affiliations:** Chicago


### ORIGINAL ARTICLES.

### Treatment of Rectal Fistula.

BY CHARLES J. DRUECK, M. D., CHICAGO.

In those few cases that you may see from time to time where the abscess has not yet ruptured, it should be freely opened away from the rectum and the cavity thoroughly and carefully drained. The incision should be made in a line radiating from the anus, as such an incision causes the least amount of hemorrhage. In the great majority of cases you will not be consulted until a fistula has formed.

Exceptionally a fistula heals spontaneously or after some slight irritation or stimulation as the passing of a probe or an injection of an irritant into it. Such a cure can occur only in a recent fistula resulting from a superficial abscess with a straight track which is lined with healthy granulations. It cannot occur after the induration develops around the sinus as is seen in old cases. Allingham says about one per cent of these cases heal spontaneously.

Palliative treatment is not very effective as far as a cure is concerned, but much may be done to relieve the suffering when the patient objects to active, operative treatment. The bowels should be kept freely open enough to allow a soft unformed movement; such a condition prevents stretching and lacerating the tissues and also prevents forcing feces into the sinus. After each bowel movement the rectum should be flushed out with an enema of plain water to keep the parts clean. If the fistula is a large one, with a free exit, it may be washed out with a piston syringe, with either plain water or a five per cent solution of carbolic acid, which will wash away the excretions and temporarily stop the seeping. All this may be attended to by the patient or his friends. The physician may occasionally use some stimulating application, as swabbing the cavity twice a week with tr. iodine or carbolic acid 95 per cent, quickly followed with alcohol to prevent any excess action. Drainage is facilitated in all cases by dilating the external opening or all of the tract with metal dilators or sea tangle tents, and then put in drainage tubes.

This also stimulates granulations by lacerations. Local treatment, however, is not sufficient, and the general health must be improved. Alteratives, tonics and digestants must be given in full doses whenever indicated.

### Operative Treatment.

As a rule, it is well to operate promptly in all cases unless there is some contra-indication. It is not well to operate upon an acutely inflamed fistula, because the peri-rectal areolar tissue is very liable to break down. In systemic complications such as cancer, Bright's disease, liver or heart trouble, an operation may be absolutely contra-indicated. The perplexing question comes with tubercular subjects. In my own practice, I operate upon tubercular patients the same as others if there is a chance of effecting a cure of the local trouble, because it relieves the patient of at least one source of waste and worry. It must be remembered also that primary tuberculosis of the rectum exists, and like a tubercular testicle, if early removed, the patient may be spared extention, and a complete cure expected. There is also some timidity in operating lest the wound in a tuberculous patient fail to heal. A properly selected case will always heal under careful general treatment. With phthisical patients it must be remembered that the vitality is low, and the operation should not be too extensive at one time for his recuperative power. Of course, no one would operate upon an active case of pulmonary tuberculosis. Where there is frequent and violent coughing it contra-indicates operation, because the movements of the rectum during coughing prevents healing. The rather common belief among the laity that the fistula is the outlet of some vicious humors which produce the consumption, if the fistula is healed, is not supported by investigation. Many operators have reported good results from operation even in advanced tuberculosis. Kelley says: "Only once have I seen the cure of a fistula followed by marked increase of the lung trouble and in that case the relationship between cause and effect cannot be established."

Whenever there is even a suspicion of tubercular disease the whole fistula should be curretted and cauterized with a Paquelin cautery or removed en masse. When there is extensive anemia it may contra-indicate operation.

Operations for fistula are divided into two classes, the tenative and the radical. Tentative operations include the injection of astringents and the several methods of dividing the tissues with a ligature. All of these methods are tedious to perform and uncertain as to their final efficiency. To be honest with your patient, you can not promise anything from these methods. The charlatan by these methods induces enough inflammation for the skin to heal over until he gets his fee, but the channel soon reopens and the patient is in the same condition as before the treatment. These methods are to be used only when the patient refuses radical operation.

### Injection.

The variety of irritants that have been used to produce inflammatory sclerosis is innumerable, and some new remedy is almost daily suggested. Iodine is altogether the best, with zinc, silver, carbolic acid and ergot following.

The injection is made with a hypodermic with a blunt needle, which should be three inches long. The injection is made in and around the sinus, the left index finger being placed over the internal opening as a plug to prevent the escape of the fluid. When injecting into the sinus a small, ordinary syringe may be used, but sufficient force must be obtained to press the fluid against all of the surfaces. Nitrate of silver or carbolic acid may be used on a probe. This method is only sometimes successful, and is more painful and longer than radical operation. It gives its best results in certain recent cases of ischiorectal abscess where there is external drainage of pus but as yet no internal opening. The patient should be confined to his bed and the opening enlarged to allow the free escape of pus. The cavity should be washed out with some solution; care being taken that no pressure of the irrigating fluid occurs within the cavity, because often the wall between the abscess and the rectum is very frail. In older cases, a pyogenic membrane develops about the abscess, and more stimulating solutions are needed, as iodine in strong solution, turpentine or caustic potassium.

### Ligation.

This is a very old method being described by Celsus, but laterly brought up by Allingham, Sr. although it is scarcely used now. The operation consists in passing a ligature of some material threaded on an eyed probe, in at the external opening, through the sinus and out at the anus. It is then drawn tightly and knotted. Heavy silk or an elastic band one-eighth of an inch in diameter may be used. If the elastic ligature is used a lead shot is pinched on the knot to prevent slipping. The ligature usually cuts its way out in a few days by pressure necrosis.

The advantages of this method are (1) no knife; (2) no bleeding; (3) no anesthetic is needed; (4) there is little pain, and (5) the advocates of this method say the patient need not be confined to his bed. As a matter of fact, however, there is no more reason why a patient should be allowed to be out of bed when the cutting is being accomplished slowly with a ligature than when performed with a knife. In either instance the fistula will heal while the subject is attending to his ordinary duties, but in both cases healing goes on much better and quicker if he is confined to his bed. Various devices have been recommended for passing the ligature through the fistula, but in my mind the best is a silver probe with an eye in it.

The objections to the ligature method are (1) more time is consumed than by the radical operation; (2) only the main sinus is treated and any other tracts are left untreated; (3) the method is unreliable because sometimes the ligature cuts out only partly, a considerable slough forms along the course of the ligature from the strangulation, and it is several days before it separates. When it does considerable hemorrhage occurs.

Dividing the parts with a thermo cautery wire is a form of ligature. It is quicker but requires an anesthetic, and is then open to many of the objections of both other operations. It is, however, applicable where there is considerable anemia and the operator is anxious to save any loss of blood even at the expense of a little longer course of after treatment.

The wire should be brought to a cherry red heat only, as that is sufficient heat to divide the tissues slowly. If too great heat is used the tissue is consumed instead of being seared; no coagula is left to occlude the vessels and hemorrhage results, thereby forfeiting the best claim of the operation.

The radical operations are incision and excision. Each has its class of cases to which it is best suited, and the surgeon must not think either method applicable in all cases. Certain stereotyped methods are described in dealing with fistula, but it must be impressed that radical cure often requires desperate surgery, and sometimes much originality or genius on the part of the operator. No one knows the pathology of a fistula until it is laid open before him.

The advantages of radical operations are (1) every sinus can be hunted out; (2) overlaping edges may be removed; (3) free drainage obtained and sepsis prevented; (4) the operation is quickly and thoroughly done.

### Incision and Division of the Parts.

The bowels should be thoroughly emptied the day previous but too great cathersis must not be provoked as a diarrhea proves a great disadvantage to the operator. Six hours before the operation a high enema is given to wash out the colon and rectum. The hair about the parts is shaved off and the whole perineum and buttocks are thoroughly scrubbed. These details are just as important as if primary union were expected. While we have an infected field and pus cavity to begin with we are also making fresh wounds, which should be as near aseptic as possible.

The patient is anesthetized, placed in the exaggerated lithotomy position, and the sphincters thoroughly paralized by overdistention. Always anesthetize the patient, even if the fistula seems small and superficial, because the operation is painful, and you can not fortell the condition of the sinus. Only the small simple fistulae may be incised without anesthesia.

The rectum may now be inspected and a final antiseptic irrigation given.

Pass a grooved director through the sinus into the rectum. It can usually be brought out at the anus and supported by the index finger of the left hand. By introducing the left index finger into the rectum before introducing the director, this maneuver is facilitated. A heavy, sharp pointed bistoury, is passed along the groove of the director and divides the bridge of tissues, thus exposing the whole tract. The sphincter should be cut squarely across as incontinence is less likely to follow than if the fibers are cut diagonally. The edges are now held apart, and a careful examination made for diverticula, which if found, should be treated as the first sinus. The scar tissue and membranous lining of the fistula should be carefully dissected away.

If the director cannot be brought out at the anus when introduced it is good surgery to dissect down upon it instead of using excessive force, as there is danger of breaking the instrument, which is a very annoying accident. Scissors are often preferable to the knife in such cases.

If the internal opening is high up, as sometimes is found in the internal incomplete, it is well to divide the anal portion of the rectum in the posterior rhaphe, even to the tip of the coccyx if need be.

If the wound is extensive and there are any parts not thoroughly drained, so called dead spaces, they should be closed with a few appropriate stitches. Free drainage is however the watchword of success in all fistula operations, and sutures are to be placed only with great care and never if the wound is superficial. After hemorrhage has been stopped the wound is packed with iodoform gauze and a suppository of opium placed in the rectum. A pad of absorbent gauze or cotton retained with a T bandage, is all the dressing required, and need not be changed until they become soiled.

The bowels should be moved on the fourth day, by giving a cathartic followed by a copious enema just before the bowels move. This brings away the packing in the wound and after defecation is accomplished, the wound should be carefully washed and another strip of gauze inserted in the base of the wound to prevent the skin edge of the incision healing before the deeper structures. If there is any tendency toward skin healing the surfaces should be separated daily by passing a probe between them. Granulation may be stimulated if need be by balsam of peru or by touching superabundant granulations with stick nitrate of silver.

Whenever there are any diverticulae, they should also be dissected out and treated as the main tract, unless there is much danger of incontinence resulting, or of injuring to a great extent the perineum in the case of women, or of overtaxing the patient's reparative powers.

The sphincter may generally be cut once, especially in the median line, without danger of incontinence, but always divide as little as possible, and leave the internal sphincter, if possible. Never divide the sphincter twice at the same operation or without informing the patient that some incontinence is sure to result. The condition of the patient's bowels, as regards the number and character of movements, must always be considered. A sphincter that acts admirably for one formed movement daily, may be totally ineffectual in case of periodical constipation and diarrhea. Division of the sphincter may cause some incontinence even in a robust man, and dilitation in a delicate woman may leave some permanent paralysis. In fistula cases, the sphincters are weak, at best, and should not be incised or stretched any more than is absolutely necessary. The liability to incontinence after division, is found chiefly in horseshoe and rectolabial varieties, and those old cases where the whole anal region is riddled with openings, but it is these cases that give the operator a chance to show his resources and ingenuity.

In the treatment of all fistulae that extend above the internal sphincter, the latter must be divided, with at least temporary incontinence. A permanent paralysis results if the sphincter is cut several times.

Where the tracts extend above the internal opening in the bowel, the extension must be considered a diverticula, and so treated. Sometimes an operator, for fear of hemorrhage, will incise only to the internal opening, and trust to inflammatory adhesion for the branch. Such treatment is risky.

The branches running under the bowel are of two varieties. One kind extends along beneath the mucous membrane and does not bleed much when cut. The other runs deeper under the muscular coat and even into the peri-rectal tissues. In these there may be considered hemorrhage. Many sini extend under the skin from the bowel to the buttock or scrotum, and these may be laid open without interfering with the sphincters.

Where there are several fistulous tracts (in old cases) the patient's reparative powers are often weak, and it is better to do two small operations than to tax the recuperative power with one extensive one. In these cases there exists often two or three main tracts apparently primary, each of which has its smaller diverticula. Each main tract and its branches may well be treated at separate operations.

When no internal opening can be found and the director moves under the thin layer of mucous membrane it is well to make an opening by forcing the director into the bowel and treat as a complete fistula. If more than one internal opening is found they should be incised into a common tract.

### After Treatment.

A few points in the after treatment of these cases are as essential as the details of the operation itself. Too frequent dressing may indefinitely postpone healing, as also packing it too tightly, and the surface assumes a membranous appearance instead of healthy granulations. Too energetic application of antiseptics also retards healing. Very often I use no dressing at all after the first packing but separate the edges every day to insure healing from the bottom and not allow lapping of the skin edges together, which is very likely to occur in the skin near the anus.

Pain is not severe enough to need opiates, and as they affect the stomach the patient is better off without them. The diet should be nourishing, soft and of such kind as to leave little residue, but must not be scanty as all these patients are anemic and need prompt building up. With this diet the bowels have no tendency to move for several days and need not be tied up.

How long the patient shall be confined to his bed depends altogether on the case. In a favorable one, he could be kept quiet until the wound heals, as moving about retards healing. Tubercular patients should keep their bed no longer than is absolutely necessary and while not allowed to move about much, they should be urged into the fresh air and sunshine, for every thing must be done to build up the system; nutritious diet, change of scenes and such medicines as are tissue builders. Locally the treatment is stimulating, but it is wrong to worry the patient with frequent dressings. Many times the wounds appear healthy but refuse to granulate. If there is no sinus to account for the sluggishness, turn your attention to the general system. If there is any evidence of pus burrowing or the formation of a pocket it should be immediately incised.

After what has been said regarding the treatment of fistula, let me say that each case is a rule to itself, and no complication can be treated according to definite procedure. Allingham says it requires more dexterous surgery to cure a bad case of fistula than any other surgical affection. Do not promise too much for the first operation, as frequently a case is better treated in two or three steps, and if the reparative powers are over-taxed granulation and healing will not take place. But if each case is carefully and conservatively treated you may expect to cure 98 per cent of them.

